# The Effects of Pertuzumab and Its Combination with Trastuzumab on HER2 Homodimerization and Phosphorylation

**DOI:** 10.3390/cancers11030375

**Published:** 2019-03-16

**Authors:** Babak Nami, Hamid Maadi, Zhixiang Wang

**Affiliations:** Department of Medical Genetics and Signal Transduction Research Group, Faculty of Medicine and Dentistry, University of Alberta, Edmonton, AB T6G 2H7, Canada; namimoll@ualberta.ca (B.N.); hmaadi@ualberta.ca (H.M.)

**Keywords:** pertuzumab, trastuzumab, breast cancer, HER2, homodimer, phosphorylation

## Abstract

Pertuzumab (Perjeta) is an anti-HER2 monoclonal antibody that is used for treatment of HER2-positive breast cancers in combination with trastuzumab (Herceptin) and docetaxel and showed promising clinical outcomes. Pertuzumab is suggested to block heterodimerization of HER2 with EGFR and HER3 that abolishes canonical function of HER2. However, evidence on the exact mode of action of pertuzumab in homodimerization of HER2 are limited. In this study, we investigated the effect of pertuzumab and its combination with trastuzumab on HER2 homodimerization, phosphorylation and whole gene expression profile in Chinese hamster ovary (CHO) cells stably overexpressing human HER2 (CHO-K6). CHO-K6 cells were treated with pertuzumab, trastuzumab, and their combination, and then HER2 homodimerization and phosphorylation at seven pY sites were investigated. The effects of the monoclonal antibodies on whole gene expression and the expression of cell cycle stages, apoptosis, autophagy, and necrosis were studied by cDNA microarray. Results showed that pertuzumab had no significant effect on HER2 homodimerization, however, trastuzumab increased HER2 homodimerization. Interestingly, pertuzumab increased HER2 phosphorylation at Y1127, Y1139, and Y1196 residues, while trastuzumab increased HER2 phosphorylation at Y1196. More surprisingly, combination of pertuzumab and trastuzumab blocked the phosphorylation of Y1005 and Y1127 of HER2. Our results also showed that pertuzumab, but not trastuzumab, abrogated the effect of HER2 overexpression on cell cycle in particular G1/S transition, G2/M transition, and M phase, whereas trastuzumab abolished the inhibitory effect of HER2 on apoptosis. Our findings confirm that pertuzumab is unable to inhibit HER2 homodimerization but induces HER2 phosphorylation at some pY sites that abolishes HER2 effects on cell cycle progress. These data suggest that the clinical effects of pertuzumab may mostly through the inhibition of HER2 heterodimers, rather than HER2 homodimers and that pertuzumab binding to HER2 may inhibit non-canonical HER2 activation and function in non-HER-mediated and dimerization-independent pathway(s).

## 1. Introduction

HER2 (ErbB2/Neu) is a 185 kDa transmembrane receptor belongs to the tyrosine kinase epidermal growth factor receptor family including other receptors EGFR (HER1/ErbB1), HER3 (ErbB3) and HER4 (ErbB4) [1,2,3] Dimerization of HER receptors leads to activation of their intracellular tyrosine kinase domains leading to the phosphorylation of both receptors [3]. Phosphorylated HER receptor dimers initiate multiple signaling pathways including PI3K/AKT, PLC-ɣ and MAPK signaling pathways, which promotes cell growth, division, and motility [3]. Activation of HER2 tyrosine kinase domain takes place after homodimerization and heterodimerization with either EGFR, HER3, or HER4. HER2 is encoded by the *ERBB2* gene which is known as an oncogene and amplification causes overexpression of HER2 receptor in the cells. Overexpression of HER2 mostly due to gene amplification is a common oncogenic phenomenon in many cancer types and is associated with poor clinical outcome [4]. HER2 is overexpressed more than 10 times in tumor cells than that in normal cells in 15–30% of all breast cancers [2,5,6,7], 2–66% of all ovarian cancers [8,9], and 4–35% of all lung adenocarcinoma [10,11]. The cancers with HER2 overexpression are known as “HER2-positive cancers”. Compared to other subtypes, HER2-positive cancers grow faster due to more HER2 signaling but are vulnerable to anti-HER2 targeting therapies including pertuzumab and trastuzumab.

Pertuzumab (originally known as 2C4 and commercially known as Perjeta^®^, Hoffmann-La Roche, Basel, Switzerland), is a fully humanized recombinant anti-HER2 monoclonal antibody. Pertuzumab is approved by FDA to be used as neoadjuvant in combination with trastuzumab (Herceptin^®^, Hoffmann-La Roche, Basel, Switzerland), another anti-HER2 monoclonal antibody, and docetaxel for the treatment of early stage and metastatic HER2-positive breast cancer [12,13,14]. Adding pertuzumab to trastuzumab and docetaxel has produced better outcome than treatment with trastuzumab and docetaxel alone, including significant improvement in progression-free and overall survival rates [15,16,17]. Binding pertuzumab to HER2 of HER2-positive tumor cells coats the tumor cells by Fc domain of the antibody that are immunogenic ligands for Fc receptor of cytotoxic immune cells. This mechanism provokes the immune cells to attack and destroy the tumor cells by releasing cytotoxic enzymes and apoptosis induction the process called antibody-dependent cellular cytotoxicity (ADCC) [18,19,20,21]. In addition to induction ADCC, pertuzumab also showed to inhibit HER2-positive cancer cell proliferation in the absence of immune cells, implicating the anti-cancer effects of the pertuzumab through alteration of HER2-mediated signaling pathways [22,23,24]. Pertuzumab binds to the dimerization pocket in the domain II of the extracellular part of HER2 that is believed to inhibit HER2/EGFR [25] and HER2/HER3 heterodimerizations [26,27,28,29]. Since the heterodimerization between HER2 and EGFR/HER3 is induced by ligand-binding, pertuzumab is believed to blocks ligand-dependent activation of HER2 and downstream signaling [25,28,29,30].

Given the better outcome of pertuzumab treatment in combination with trastuzumab, there seems to be a synergism between the two therapeutics [31]. Trastuzumab binds to extracellular domain IV close to the transmembrane region of HER2 [12,32]. Trastuzumab is reported to block the homodimerization of HER2, and to inhibit ligand-independent HER2-mediated signaling as HER2 is an orphan receptor, but could homodimerize when overexpressed [31,33,34]. However, we previously showed that trastuzumab does not inhibit HER2 homodimerization, phosphorylation and downstream signaling [35]. So far evidences on exact mode of action of pertuzumab, particularly its role in blocking HER2 homodimerization, HER2-mediated cell cycle progression and cell death still remains controversial. In present study we investigated the effects of pertuzumab and its combination with trastuzumab on homodimerization and tyrosine phosphorylation of HER2 as well as on the gene expression in HER2 overexpressing cell line model.

## 2. Results

### 2.1. Specific Binding of Pertuzumab to HER2

**Figure 1:**
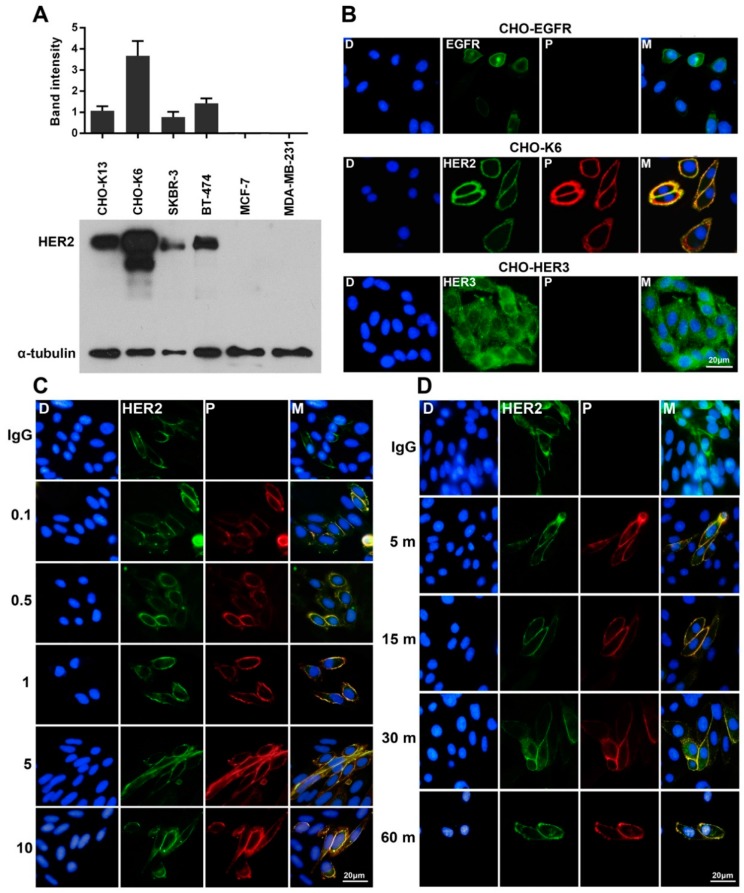
Specific binding of pertuzumab to HER2. (**A**) The expression levels of HER2 in CHO cell lines stably overexpressing HER2 (CHO-K13 and CHO-K6), two HER2-positive (SKBR-3 and BT-474) and two HER2-negative (MCF-7 and MDA-MB-231) breast cancer cell lines. (**B**) Double-immunofluorescence staining of EGFR, HER2, and HER3 (green) and pertuzumab (P; red) in CHO-EGFR, CHO-K6, and CHO-HER3 cell lines. Pertuzumab binds specifically to HER2 but not to EGFR and HER3. (**C**,**D**) Double-immunofluorescence staining of HER2 (green) and pertuzumab (red) in the cells (**C**) treated with 0.1, 0.5, 1, 5, and 10 μg/mL pertuzumab for 60 min (dose-course treatment) and (**D**) treated with 10 μg/mL pertuzumab for 5, 15, 30, and 60 min (time-course treatment). Treatment with 10 μg/mL of human IgG for 60 min was used as a mock control.

In this study we used Chinese hamster ovary (CHO) cells stably expressing human HER2 (HER2-K6 [35,36]) as HER2 overexpressing cell model. The expression level of HER2 in CHO-K6 cells was detected significantly higher than that of breast cancer cell lines including SKBR-3, BT-474, MCF-7, and MDA-MB-231, as well as another clone of HER2-overexpressing CHO cell line HER2-K13 cells [35,36] (Figure 1A). To examine binding of pertuzumab to HER receptors, we treated CHO cells stably overexpressing EGFR (CHO-EGFR), HER2 (CHO-K6) and HER3 (CHO-HER3) with 10 µg/mL pertuzumab for 60 min. As shown in Figure 1B, pertuzumab specifically bound to HER2 in cell membrane, but not to EGFR and HER3. Dose-response experiments (0.1, 0.5, 1, 5, and 10 µg/mL for 60 min) indicated that pertuzumab strongly bound to HER2 even at a low concentration of 0.1 µg/mL (Figure 1C). Moreover, time-course (10 µg/mL for 5, 15, 30, and 60 min) treatment of CHO-K6 cell showed that 5 min incubation is enough to result strong binding between pertuzumab and HER2 (Figure 1D). Together, these results confirm that pertuzumab specifically binds to HER2 receptor with high affinity.

### 2.2. The Effects of the Pertuzumab on the HER2 Homodimerization

**Figure 2:**
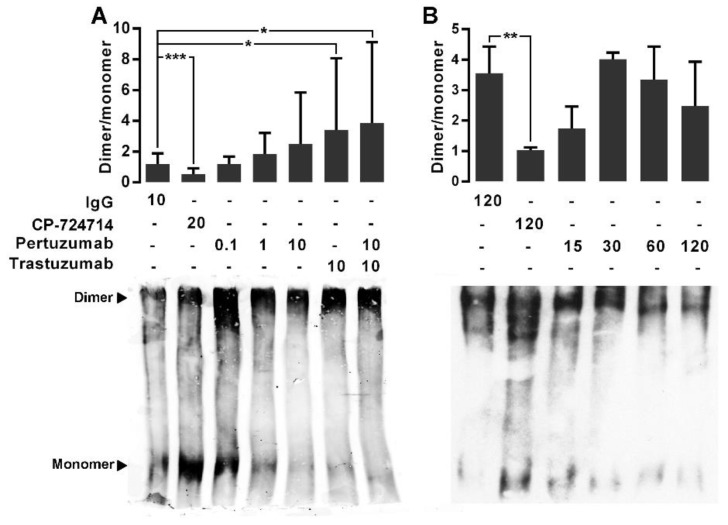
The effect of pertuzumab on HER2 homodimerization. (**A**) Immunoblot expression of monomer (185 kDa) and dimer (360–380 kDa) HER2 and quantified dimer/monomer ratios in CHO-K6 cells treated with 0.1, 1 and 10 μg/mL pertuzumab, 10 μg/mL trastuzumab or their combination for 60 min (dose-course treatment). (**B**) Time-course treatment of CHO-K6 with 10 μg/mL pertuzumab for 15, 30, 60 and 120 min. Ten μg/mL human IgG and 20 μM CP-724714 were used as mock and HER2 inhibitor controls. CP-724714 reduced dimer HER2. Trastuzumab increased dimer HER2 and pertuzumab had no significant effect on HER2 homodimerization. * *p* < 0.05, ** *p* < 0.01, *** *p* < 0.001.

We next examined the effects of pertuzumab, trastuzumab and their combination on the homodimerization of HER2. We previously showed the specific binding of trastuzumab to HER2 [35]. For dose-response experiments, the CHO-K6 cells were treated with 0.1, 1, and 10 µg/mL pertuzumab, trastuzumab, or their combination for 60 min. For time-course treatment the cells were treated with 10 µg/mL pertuzumab for 15, 30, 60, and 60 min. Ten µg/mL human IgG and 20 µM HER2 tyrosine kinase inhibitor agent CP-724714 were used as mock and positive controls respectively. HER2 monomer and homodimer was assessed by BS^3^-based protein cross-linking assay. As result, the mean ratio of quantified HER2 homodimer to HER2 monomer in the cell treated with CP-724714 was under 1, significantly lower than that of the cells treated with IgG in the dose-response experiments (Figure 2A) and the time-course experiments (Figure 2B). This indicates higher level of monomer HER2 than homodimer HER2, confirming that CP-724714 significantly inhibited HER2 homodimerization. In contrast, there was no significant difference between HER2 dimer/monomer ratio of IgG treated cells and that of the cells treated with single-agent pertuzumab in both dose- and time-course treatment experiments. While, the cells treated with trastuzumab as well as those treated with combination of pertuzumab and trastuzumab showed higher HER2 dimer/monomer ratios compared to IgG treated cells (Figure 2A). These results implicate that pertuzumab had no considerable effect on HER2 homodimerization. However, trastuzumab increased HER2 homodimerization, which is consistent with our previous observation [35].

### 2.3. The Effects of the Pertuzumab on the HER2 Phosphorylation

We have shown previously that HER2 is strongly phosphorylated in CHO-K6 cells [35]. Here, to examine whether pertuzumab inhibits HER2 phosphorylation, we investigated the phosphorylation of seven pY sites on HER2 C-terminal including Y1005, Y1112, Y1127, Y1139, Y1196, Y1221/1222, and Y1248. CHO-K6 cells were treated with pertuzumab at final concentrations of 0.1, 1 and 10 µg/mL, 10 µg/mL trastuzumab, and a combination of 10 µg/mL pertuzumab and trastuzumab for 60 min, and then the levels of total HER2 and the phosphorylated HER2 were analyzed by Western blotting. A final concentration of 10 µg/mL human IgG and 20 µM CP-724714 were used as mock and positive controls, respectively. 

**Figure 3:**
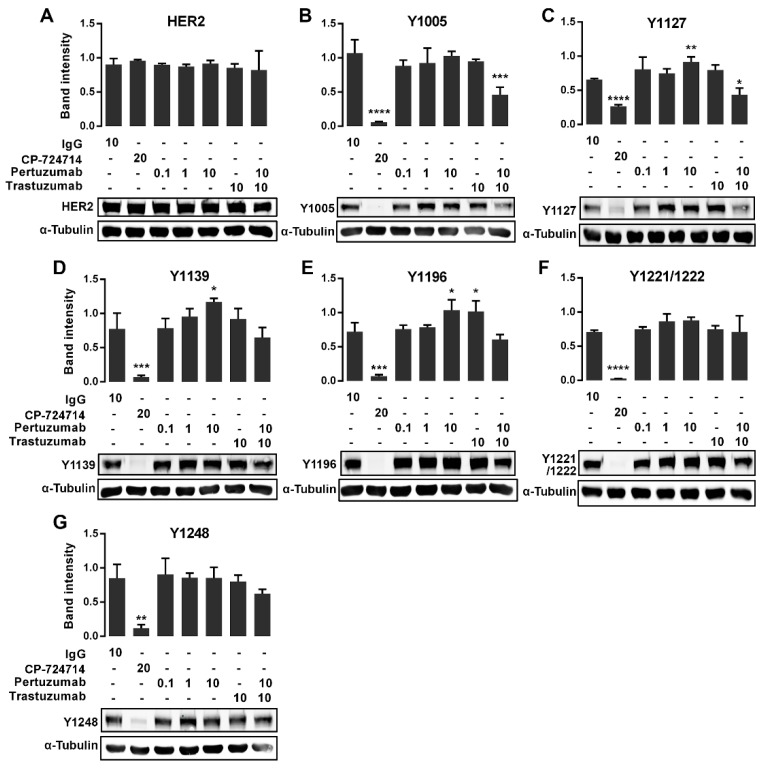
Immunoblot expression of pHER2 in pertuzumab treated CHO-K6 cells. CHO-K6 cells were treated with 0.1, 1 and 10 μg/mL pertuzumab, 10 μg/mL trastuzumab and their combination for 60 min. Ten μg/mL human IgG and 20 μM CP-724714 were used as mock and positive controls respectively. The expression of (**A**) total HER2, (**B**) pY1005, (**C**) pY1127, (**D**) pY1139, (**E**) pY1196, (**F**) pY1221/1222 and (**G**) pY1248 HER2 was monitored by immunoblotting. * *p* < 0.05, ** *p* < 0.01, *** *p* < 0.001, **** *p* < 0.0001.

As shown in Figure 3A, none of treatments showed change in the protein expression levels of total HER2. However, CP-724714 dramatically reduced the phosphorylation of HER2 at all the pY sites (Figure 3). Surprisingly, the cells treated with 10 µg/mL pertuzumab showed an increased level of HER2 phosphorylation at Y1127 (Figure 3C), Y1139 (Figure 3D) and Y1196 (Figure 3E), but not at other pY sites. On the other hand, treatment with 10 µg/mL trastuzumab only increased HER2 phosphorylation at pY1196 (Figure 3E), but not at any other pY sites. More interestingly, the treatment with combined pertuzumab and trastuzumab reduced the phosphorylation of HER2 at pY1005 (Figure 3B) and pY1127 (Figure 3C). 

**Figure 4:**
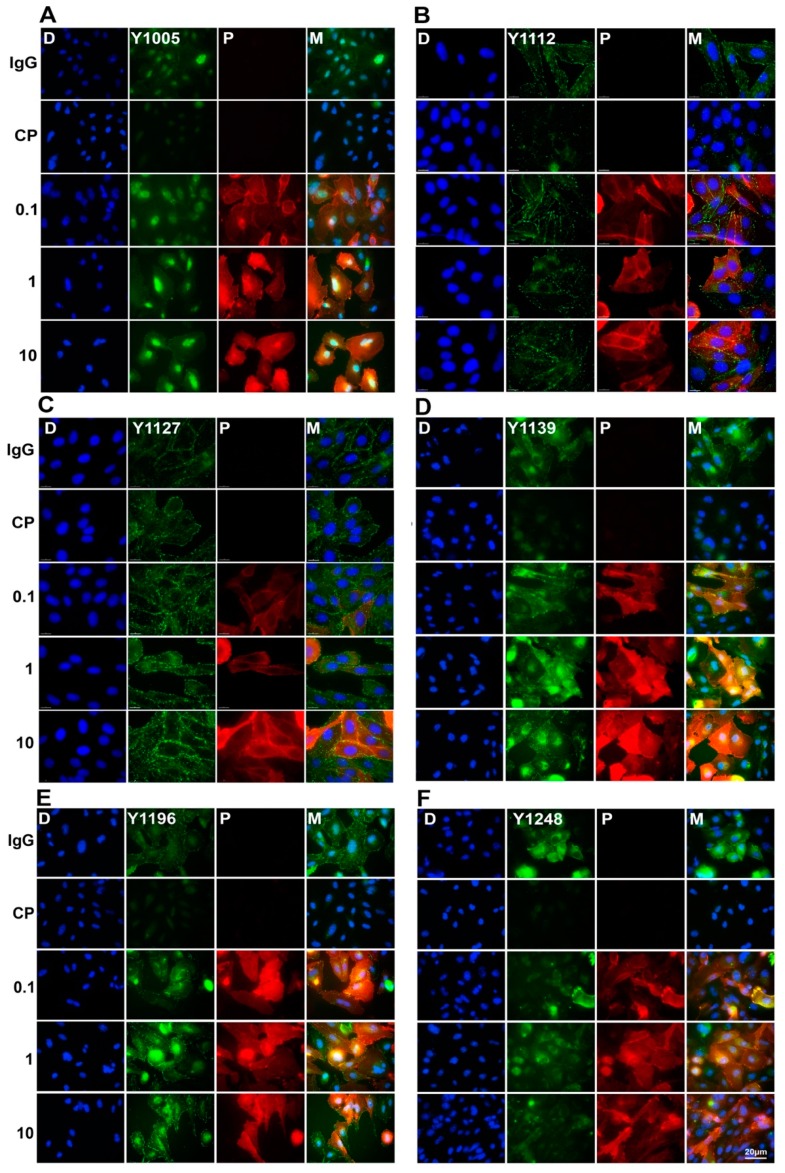
Double-immunofluorescence staining of pHER2 and pertuzumab in pertuzumab treated CHO-K6 cells. CHO-K6 cells were treated with 0.1, 1 and 10 μg/mL pertuzumab for 60 min then pHER2 at (**A**) pY1005 (**B**) pY1112, (**C**) pY1127, (**D**) pY1139, (**E**) pY1196, and (**F**) pY1248 HER2 (all green) and pertuzumab (red) were stained. Ten μg/mL human IgG and 20 μM CP-724714 (CP) were used as mock and HER2 positive controls, respectively.

The effects of pertuzumab on the phosphorylation of HER2 were further examined by immunofluorescence staining. CHO-K6 cell were treated with 0.1, 1, and 10 µg/mL pertuzumab for 60 min. The phosphorylation of HER2 at Y1005, Y1112, Y1122, Y1139, Y1196, and Y1248 was examined by immunofluorescence staining with specific antibodies as indicated. As shown in Figure 4, CP-724714 treatment dramatically decreased HER2 phosphorylation at all the pY sites. Similar to the Western blotting results, pertuzumab increased HER2 phosphorylation at pY1127 (Figure 4C), pY1139 (Figure 4D) and pY1196 (Figure 4E) compared to IgG treated cells. Pertuzumab had no significant effects on HER2 phosphorylation at other pY sites (Figure 4). Taken together, pertuzumab did not inhibit HER2 phosphorylation, but induced phosphorylation of HER2 at Y1127, Y1139, and Y1196 residues.

### 2.4. The Effect of Pertuzumab on Gene Expression Profile of HER2 Overexpression Cells

**Figure 5:**
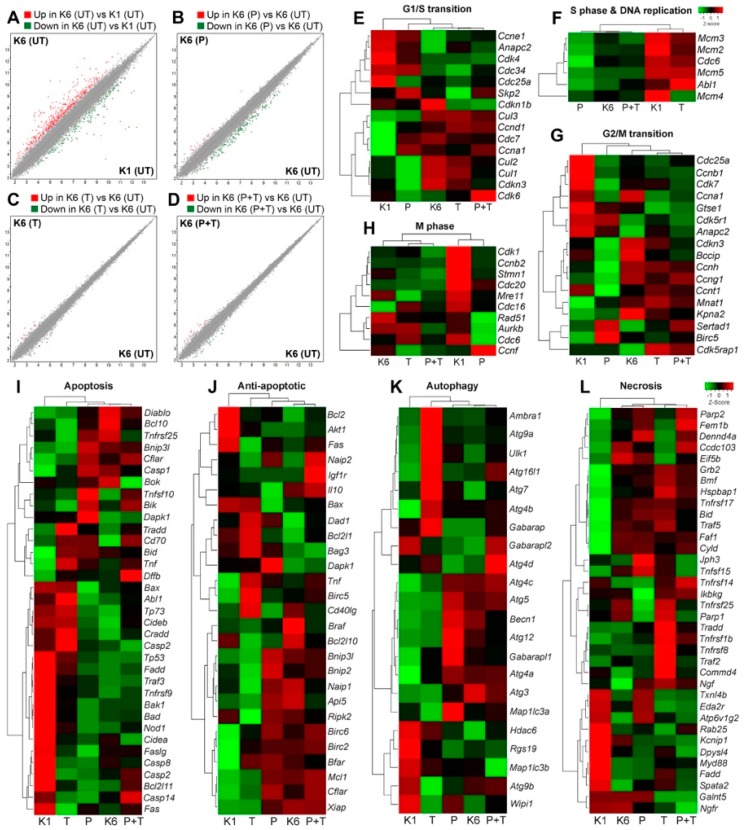
The effects of pertuzumab on gene expression profile of HER2 overexpressing cells. The whole transcript levels were evaluated by microarray for untreated CHO-K1, untreated CHO-K6 and CHO-K6 treated with 10 μg/mL pertuzumab (P), 10 μg/mL trastuzumab (T), and their combination (P+T) for 24 h. (**A**–**D**) Scatter plot showing the whole gene expression profile of (**A**) untreated CHO-K6 (K6) vs. untreated CHO-K1 (K1) cell lines, (**B**) pertuzumab treated vs. untreated CHO-K6 cells, (**C**) trastuzumab treated vs. untreated CHO-K6 cells and (**D**) CHO-K6 cells treated with the combination of the monoclonal antibodies vs. untreated cells. The values with a minimum of two-fold change are illustrated in red (upregulated) and green (downregulated) dots. (**E**–**L**) Hierarchal heatmap illustrating z-score expression of selected marker genes for (**E**) G1/S transition, (**F**) S phase and DNA replication, (**G**) G2/M transition, (**H**) M phase of cell cycle, (**I**) apoptosis, (**J**) anti-apoptosis, (**K**) autophagy, and (**L**) necrosis.

To investigate the effect of pertuzumab on gene expression profile of HER2 overexpressing cells, we treated CHO-K6 and parental CHO (CHO-K1) cells with 10 µg/mL pertuzumab, or 10 µg/mL trastuzumab for 24 h and then examined the whole transcriptome by microarray analyzing 30,934 unique cDNA. Results showed a minimum of two-fold changes in the levels of totally 606 (1.96%) transcripts between CHO-K6 and CHO-K1 cells. Of the 606 transcripts, 427 (1.38%) and 179 (0.58%) transcripts were respectively upregulated and downregulated in the CHO-K6 cells compared to the CHO-K1 cells (Figure 5A). Comparison of the pertuzumab treated with the untreated CHO-K6 cells revealed a minimum of 2-fold changes in the levels of 171 (0.55%) transcripts, of which the expression levels of 19 (0.06%) and 152 (0.49%) transcripts were respectively upregulated and downregulated in the result of pertuzumab treatment (Figure 5B). In the CHO-K6 cells treated with trastuzumab the expression of 27 (0.09%) transcripts were altered, of which 14 (0.05%) and 13 (0.04%) transcripts were respectively upregulated and downregulated compared to the untreated cells (Figure 5C). Treatment with a combination of pertuzumab and trastuzumab resulted in altered expression of 35 (11%) transcripts including 10 (0.03%) upregulated and 25 (0.08%) downregulated transcripts in the cells compared to the untreated cells (Figure 5D). These results suggest that overexpression of HER2 and in CHO cells dramatically changes the gene expression profile the cells. Additionally, both pertuzumab and trastuzumab have major effects on gene expression profile of CHO-K6 cells.

### 2.5. The Effect of Pertuzumab on Cell Cycle Progression

To examine the effect of pertuzumab on the cell cycle we analyzed the expression of the genes that are highly expressed in each stage of cell cycle in CHO-K6 cells treated with pertuzumab, trastuzumab, and their combination. Result showed significant difference between untreated parental CHO (CHO-K1) cells and untreated CHO-K6 cells regard the expression of G1/S transition, S phase and DNA replication, G2/M transition and M phase marker genes (Figure 5E–H). These most likely due to overexpression of HER2 in CHO-K6 cells. The expression levels of G1/S transition marker genes in the pertuzumab treated CHO-K6 cells were different from those of untreated CHO-K6 cells but similar to those of CHO-K1 cells (Figure 5E). However, trastuzumab treated CHO-K6 cells was similar to untreated CHO-K6 cells in terms of the expression of G1/S transition marker genes. These results indicate that pertuzumab but not trastuzumab abrogated the effect of HER2 overexpression on the expression of G1/S transition marker genes. Interestingly, the expression pattern of G1/S transition marker genes in CHO-K6 cells treated with the combination were very similar to those in untreated as well as trastuzumab treated CHO-K6 cells (Figure 5E).

CHO-K6 cells showed lower expression levels of S phase marker genes compared with CHO-K1 cells, which suggests that HER2 overexpression inhibits the expression of S phase marker genes. Treatment of CHO-K6 cells with pertuzumab did not change the expression profile of the S phase marker genes. In contrast, treatment of CHO-K6 cells with trastuzumab stimulated the expression levels of the S phase marker genes, making similar to those of CHO-K1 cells. Moreover, the expression levels of S phase marker genes in CHO-K6 cells treated with combination of pertuzumab and trastuzumab were low and similar to those of untreated as well as pertuzumab treated cells (Figure 5F). These results indicate that trastuzumab, but not pertuzumab was able to abrogate the effect of HER2 overexpression on the expression of the S phase marker genes. 

The expression levels of G2/M transition marker genes in CHO-K1 and CHO-K6 was different, which suggests the role of HER2 overexpression. Pertuzumab changed the expression of some G2/M transition marker genes in CHO-K6 cells. The expression of G2/M transition marker genes in trastuzumab treated cells was very similar to that of untreated CHO-K6 (Figure 5G). 

Similarly, HER2 overexpression also affect the expression profile of the M phase marker genes as there were significant differences between CHO-K1 and CHO-K6 cells in terms of the expression of M phase marker genes. Hierarchical clustering showed resemblance between the expression profile of M phase marker genes in the untreated CHO-K6, trastuzumab treated and the cells treated with combination of trastuzumab and pertuzumab (Figure 5H), whereas the expression of M phase marker genes in pertuzumab treated CHO-K6 cells was similar to that of CHO-K1 cells, however, this similarity was not significant (Figure 5H). Taken together, in CHO-K6 cells, pertuzumab abrogated the effect of HER2 overexpression on the expression of G1/S transition, G2/M transition, and M phase genes. Whereas, trastuzumab inhibited the HER2 effect on S phase genes. More interestingly, the effect of combination treatment of pertuzumab and trastuzumab was different compared to the effect of treatment with single agent pertuzumab or trastuzumab on cell cycle of CHO-K6.

### 2.6. The Effect of Pertuzumab on Cell Death Pathways

To study the effect of the pertuzumab on cell death pathways we investigated the expression of selected marker genes for apoptosis, autophagy and necrosis in the cells treated with pertuzumab, trastuzumab, or their combination. Similar to the cell cycle marker genes, there was opposite expression profiles of cell death marker genes between CHO-K1 and CHO-K6 cells, implicating the significant effects of HER2 overexpression on the cell death pathways (Figure 5I–L). The expression profiles of apoptotic (Figure 5I), anti-apoptotic (Figure 5J), autophagy (Figure 5K) and necrosis (Figure 5L) marker genes in pertuzumab treated CHO-K6 cells were similar to those in untreated CHO-K6, which suggests that pertuzumab does not inhibit HER2 function in regulating cell apoptosis. In contrast, trastuzumab inhibited the function of HER2 in regulating cell apoptosis as on apoptosis and anti-apoptosis gene expression profiles of CHO-K6 cells treated with trastuzumab were very different from untreated CHO-K6 cells, but similar to CHO-K1. Similar results were observed for the expression profile of autophagy and necrosis marker genes (Figure 5J–L). Furthermore, the CHO-K6 cells treated with the combination of trastuzumab and pertuzumab were similar to untreated CHO-K6 cells in the point of view of all the cell death expression profiles (Figure 5I–J). Taken together, the results suggest that pertuzumab treatment did not have significant effects on the cell death pathways of CHO-K6 cells. However, trastuzumab treatment inhibited the expression of apoptosis, but not autophagy and necrosis marker genes in CHO-K6 cells.

### 2.7. The Effect of Pertuzumab on Cell Proliferation

We next investigated the effects of pertuzumab on the cell proliferation in CHO-K6 cells. We cultured CHO-K1, CHO-K6, CHO-K13, CHO-EGFR and CHO-HER3 cells in the presence of 10 µg/mL pertuzumab or 10 µg/mL trastuzumab or their combination for 72 h and then evaluated viable cells by MTT assay. Human IgG (10 µg/mL) was used as mock control for the monoclonal antibodies. CP-724714 (10 µM) was used as positive control of HER2 inhibition. Paclitaxel (5 µM) was used as positive control of cell proliferation inhibition.

**Figure 6:**
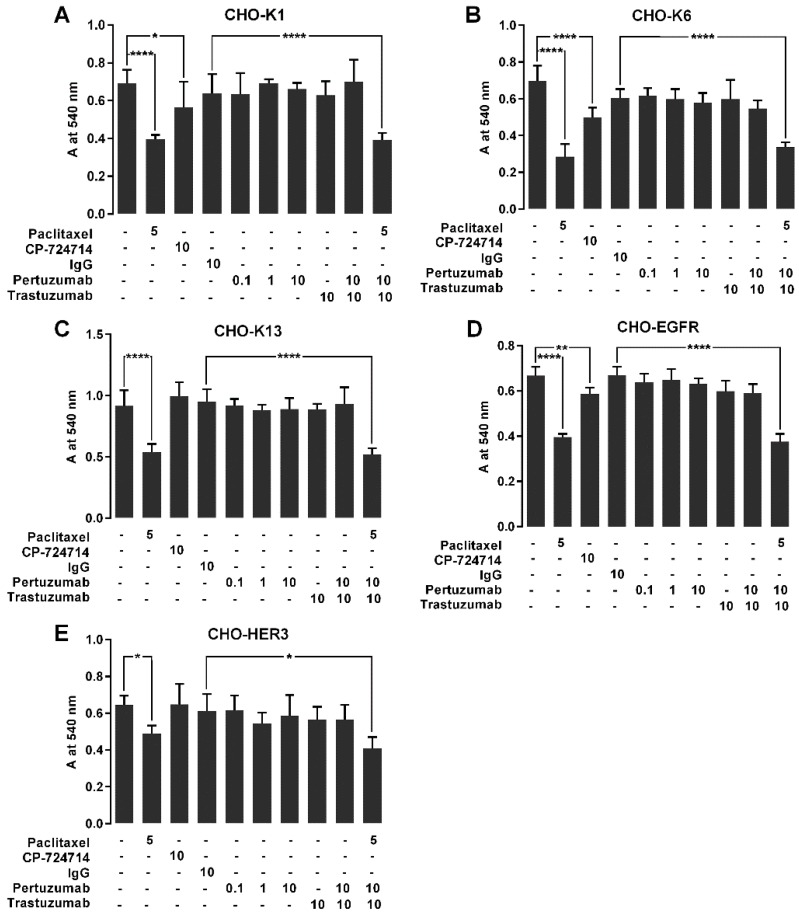
The effect of pertuzumab on the proliferation of cells overexpressing EGFR, HER2 and HER3. (**A**) CHO-K1, (**B**) CHO-K6, (**C**) CHO-K13, (**D**) CHO-EGFR and (**E**) CHO-HER3 cell lines were treated with 0.1, 1 and 10 μg/mL pertuzumab, 10 μg/mL trastuzumab and their combination for 72 h, and then the living cell mass was evaluated by MTT assay. Five µM paclitaxel, 10 μg/mL human IgG and 20 μM CP-724714 were used as respectively antiproliferative, mock and HER2 inhibitor controls. * *p* < 0.05, ** *p* < 0.01, **** *p* < 0.0001.

The results showed that the proliferation rates of all the paclitaxel treated cell lines were significantly lower than that of relevant untreated cells. Among the cell lines treated with CP-724714, CHO-K1, CHO-K6 and CHO-EGFR but not CHO-K13 and CHO-HER3 cells showed lower proliferation rates compared to the relevant untreated cells (Figure 6). None of the cells treated with pertuzumab, trastuzumab, and their combination showed significant changes in the proliferation rates in comparison with relevant IgG treated cells (Figure 6). These results indicate that treatment with pertuzumab, trastuzumab and their combination did not have significant effect on the proliferation of HER2, HER3, and EGFR overexpressing CHO cells. 

## 3. Discussion

In this study we investigated the effect of pertuzumab on the function of HER2 homodimers. We also compared the effects of pertuzumab with trastuzumab and the combination of pertuzumab and trastuzumab. Three CHO cell lines stably overexpressing human HER2 (CHO-K6), human EGFR (CHO-EGFR) and human HER3 (CHO-HER3) as well as parental CHO (CHO-K1) cells were used in our study. The parental CHO cell line dose not express any of HER family receptors per se. CHO-K6 provides appropriate model cell to study HER2 receptor function that allows monitor HER2 function without interaction by other HER receptors. Although pertuzumab specifically binds only to HER2, studying HER2 homodimerization and its subsequent effects on the cellular biology is not quite feasible in breast cancer cell lines. Since HER2-positive breast cancer cell lines express also at least one other member of HER receptor family particularly EGFR and HER3 [37,38,39]. Endogenous EGFR and HER3 could mediate in HER2 heterodimerization and significantly affect the HER2 phosphorylation. On the other hands, the CHO cell lines allow us to study each HER receptor independently. CHO-K6 cells show high rates of HER2 homodimerization and phosphorylation that can be inhibited by CP-724714. Despite canonical downstream signaling pathways of HER2 (PI3K/AKT and MAPK pathways) do not work in CHO-K6 cells, the cell proliferation rate is higher than that of parental CHO cells (CHO-K1) [35]. This HER2-mediated increased proliferation can be inhibited by lapatinib and CP-724714. Moreover, we showed that the monoclonal antibodies changed the gene expression profile of the cells. These conform that CHO-K6 cell line is sensitive to anti-HER2 agents. This sensitivity is revealed by inhabitation of HER2 dimerization, phosphorylation and CHO-K6 cell proliferation in response to treatment with the agents. These evidences further confirm that CHO-K6 is a suitable cell model for studying HER2 homodimerization and phosphorylation but is not an appropriate model for studying HER2-mediated PI3K/AKT and MAPK pathways. Furthermore CHO-K6 cells provides a valuable cell model for studying the oncogenic function of HER2 via non-canonical mechanism(s) independently of PI3K/AKT and MAPK signaling pathways that deserves paying more attention. 

We showed that pertuzumab specifically bound to HER2 with high affinity on CHO-K6 cells. No pertuzumab binding was detected on CHO-EGFR and CHO-HER3 cells. Pertuzumab binds at the dimerization pocket of HER2 located in its extracellular domain II [27], while trastuzumab binds to a site located in extracellular domain IV [40]. Thus, it is suggested that the binding of pertuzumab but not trastuzumab to HER2 disrupts its heterodimerization with EGFR [25] and HER3 [26,27,28,29,31]. However, there is no independent research focusing on the effect of pertuzumab on HER2 homodimerization, phosphorylation and gene expression profile. Structurally, conformation of monomer HER2 is resemble to ligand-bound EGFR receptor. Therefore HER2, when overexpressed, is able to form homodimer in the absence of ligand, which also resulted in the phosphorylation of HER2 [27,35,40]. In the present study, HER2 was highly dimerized in CHO-K6 cells, most likely due to the overexpression. We showed that pertuzumab does not have a significant effect on HER2 homodimerization, while trastuzumab and the combination of pertuzumab and trastuzumab increase HER2 homodimerization. Homodimerization of HER2 takes place through interaction of domain II of one HER2 receptor with the C-shaped pocket formed by domain I, II, and III of the adjacent HER2 receptor [41]. A previous study shows that pertuzumab but not trastuzumab inhibits HER2 homodimerization and increases the antiproliferative effect of trastuzumab on HER2-positive breast cancer cells in combination with trastuzumab [42]. In another study Hu et al. [41] report that pertuzumab binding to its epitope on domain II of HER2 prevents interaction of C-shaped pocket from adjacent HER2 with dimerization arm of HER2 by masking the dimerization pocket [41]. According to this study, pertuzumab abolishes HER2 homodimerization in COS-7 cell expressing extracellular domains of HER2, however, trastuzumab had a negligible effect on the HER2 homodimerization [41]. However, our results do not support these reports. We found that pertuzumab has no significant effect on HER2 homodimerization. In contrast, trastuzumab and the combination treatment increase homodimerization of HER2. In addition, pertuzumab is not able to abrogate the positive effect of trastuzumab on HER2 homodimerization. There are some reasons that show our results are more reliable compared to previous opposite results. Firstly; in this study we used an originally HER2-negative cell line that stably expresses full-length human HER2, while Hu et al. [41] used a partial extracellular domain of HER2 (residues 1–624). It is quite possible that partial HER2 has a structure distinct from the structure of full-length HER2. Aberrant conformation of partial HER2 most likely effects the binding and function of the monoclonal antibody on HER2. Second; Diermeier-Daucher et al. [42] used BT-474 and SKBR-3 cell lines to study homodimerization. Both of the cell lines express EGFR, HER3 and HER4 in addition to HER2. The HER receptors have similar molecular size and can form 10 different dimers with each other. Distinction of HER2 homodimer from heterodimers and homodimers of the other three HER receptors based on molecular weight may be highly erroneous. 

We showed that trastuzumab increases HER2 homodimerization, which is consistent with our previous results [35]. The epitope of trastuzumab is located near transmembrane domain of HER2 [40]. Several experimental and molecular dynamics simulation studies support the critical role of transmembrane and juxtamembrane domains in HER2 homo- and heterodimerization [43,44,45,46,47,48,49,50,51]. HER2 transmembrane has two GxxxG-like motifs, one in the N-terminal close to the extracellular domain and one in the C-terminal close the intracellular domain [45,50,51]. The N-terminal GxxxG-like motif mediates in heterodimerization, whereas the C-terminal motif had a role in the formation of homodimer [51]. It has been shown that a mutation at valine 664, such as Val664Glu which is located in the transmembrane domain between the GxxxG-like motifs, leads to constitutive HER2 activation by enhancing the tendency to dimerize [52]. Moreover, substitution of isoleucine 655 of the HER2 transmembrane with valine is found to increase breast cancer risk. This mutation changes the conformation of the receptor that causes constitutive activation of HER2 tyrosine kinase domain [53]. Further, phosphorylation of threonine 677 in the juxtamembrane domain of HER2 is shown to inhibit HER2/EGFR heterodimerization [54]. This evidence demonstrates that conformation change in the HER2 transmembrane can alter HER2 receptor dynamics resulting in an altered tendency for dimerization. Indeed, binding of trastuzumab [40] and pertuzumab [27] significantly changes HER2 conformation. It is possible that somehow trastuzumab binding confers a new conformation that facilitates homodimerization of HER2, a function that pertuzumab is not able to perform. 

We showed that pertuzumab did not inhibit HER2 phosphorylation but induced phosphorylation of HER2 at Y1127, Y1139, and Y1196 residues. Trastuzumab did not have a significant effect on HER2 phosphorylation except at Y1196. This result is consistent with our previous report that shows trastuzumab does not affect HER2 phosphorylation at any pY sites [35]. We showed that combination of pertuzumab and trastuzumab inhibits pY1005 and pY1127. Lack of inhibitory effect of pertuzumab on phosphorylation is expected since it had no inhibitory effect on dimerization. The function of pY1127 is not described yet. pY1005 have been shown to bind SHC [55]. pY1139 is an important pY for HER2 function. pY1139 is a binding site for EGFR and PI3K [56,57]. Phosphorylation of Y1139 activates RAS through GRB2 and increases transcriptional activity of STAT3 [58,59]. Phosphorylation of Y1196 enhances activation of ERK through a RAS-independent pathway and increase the binding affinity of HER2 to CRK which is a member of an adapter protein family. CRK is required for HER2 to increase RAC-dependent cell motility and HER2-mediated inhibition of apoptosis [60,61,62]. It is interesting to notice that pertuzumab did not change the phosphorylation level of HER2 Y1248. We did not observe any significant effect of trastuzumab on pY1248 in CHO-k6 cells in our previous research [35]. However, two recent studies indicate that trastuzumab increases the phosphorylation level of pY1248 in several breast cancer cell lines, and the enhanced pY1248 phosphorylation is linked to the inhibitory role of trastuzumab on cell proliferation [63,64]. 

Phosphorylation of HER2 at canonical pY sites takes place by HER receptor tyrosine kinases following homo- and heterodimerization. HER2 is not phosphorylated only by HER family receptor tyrosine kinases, but it can be also phosphorylated by other kinases in a dimerization-independent manner at tyrosine residues. For example, SRC phosphorylates HER2 at tyrosine 877 located in the P-loop of the kinase domain and increases the kinase activity of HER2 [65]. It is possible that pertuzumab has a ligand-like function that confers HER2 a new conformation providing Y1127, Y1139, and Y1196 residue binding sites for non-HER family tyrosine kinases. However, there is no evidence yet supporting or abrogating this hypothesis. Taken together we suggest that pertuzumab may have roles in non-canonical HER2 activation in a dimerization-independent manner. To test this hypothesis, the effect of pertuzumab and trastuzumab on phosphorylation of non-canonical HER2 phospho-sites and the identity of potential kinases involved in pertuzumab-induced phosphorylation will be investigated in the future research. 

Our results showed that HER2 overexpression altered the expression levels of 606 different transcripts (1.96% of all analyzed transcripts) in the CHO cell line. Most of the altered transcripts (N = 427; 70.46%) was upregulated. These numbers are enough significant to conform the huge impact of HER2 overexpression on cellular biology. A microarray study analyzing 5184 unique transcripts in HER2 overexpressing breast cancer cells and tumors revealed different expression levels of 136 (2.62%) and 151 (0.03%) transcripts in respectively HER2 overexpressing cell line and HER2-positive tumor tissues compared to low HER2 levels cell lines and tumors [66]. In another analysis of 6000 cDNA array, expression of 61 (1.02%) transcripts were found altered due to overexpression of HER2 [67]. In our study, pertuzumab treatment changed the levels of 171 transcripts in CHO-K6 that most of them (*N* = 152; 88.89%) were downregulated. However, trastuzumab only altered the levels of 27 transcripts of which 14 were up- and 13 were downregulated. The different effects of pertuzumab and trastuzumab may be due to different mode of action of these two monoclonal antibodies. 

We also showed that expression profiles of G1/S transition, G2/M transition and M phase marker genes in pertuzumab treated HER2 overexpressing cells were resemble to those of HER2-negative cells. This is a strong evidence confirming that pertuzumab inhibits HER2-mediated cell cycle progression. However, pertuzumab does not induce cell death pathways including apoptosis, autophagy and necrosis. In contrast, trastuzumab has no major effect on cell cycle but induces apoptosis. These results are supported by numerous previous reports that show pertuzumab inhibits cell cycle progression but not apoptosis and trastuzumab affects as vice versa [22,68,69,70,71]. HER2 kinase inhibitors lapatinib and CP-724714 significantly inhibit CHO-K6 cell proliferation, however, we did not observe inhibitory effect of pertuzumab and trastuzumab on the cell proliferation. This is probably because pertuzumab was unable to abrogate the HER2 effects on the cell cycle progression completely. Basically, CHO-K6 cell line grows faster than CHO-K1 cell line, due to the positive effect of HER2 overexpression on cell cycle progression of CHO-K6 cells [35]. As discussed above, the gene expression profile of almost all the 48 cell cycle regulators between CHO-K6 and CHO-K1 cell lines was considerably opposite. Pertuzumab treatment caused change in the expression levels of several cell cycle regulators but not all of them and had no major effect on some other important cell cycle regulators. For example, the expression levels of the G1/S transition regulator genes *Ccna1*, *Ccnd1*, *Cdc7*, and the G2/M transition regulator genes *Sertad1* and *Birc5* in pertuzumab-treated CHO-K6 cells were still higher than those of CHO-K1 cells. Additionally, compared to CHO-K1 cells, the expression levels of the G1/S transition regulator gene *Cdc25a*, the G2/M transition regulator genes *Cdc25a*, *Ccnb1*, *Cdk7*, and the M phase regulator genes *Cdk1*, *Ccnb2*, *Stmn1*, and *Cdc16* were still lower in pertuzumab treated CHO-K6 cells similar to untreated CHO-K6 cells. Probably, regulation of these genes by HER2 is sufficient for induction of cell cycle progression. Furthermore, this result shows that HER2 affects cell cycle progression and gene expression through different pathways which pertuzumab can block. Meanwhile, we observed that CP-724714 inhibited the proliferation of CHO-K6 and CHO-EGFR cells. This effect is correlated with the inhibitory effect of CP-724714 on HER2 dimerization and phosphorylation. This results strongly support the notion that kinase activity of HER2 is critical for its stimulatory effects on cell cycle particularly via its canonical pathways. Despite the minor inhibitory effect of pertuzumab on HER2 homodimer-mediated cell cycle progression, lack of inhibitory effects of pertuzumab and trastuzumab on cell proliferation is likely due to their inability to inhibit HER2 phosphorylation. Surprisingly, treatment with combination of pertuzumab and trastuzumab showed distinct effect on the expression of cell cycle and death marker genes compared to single agent treatment. This result suggests that not only binding of single agent pertuzumab and trastuzumab, but dual binding of the monoclonal antibodies to HER2 have unique effects on HER2-mediated cell cycle progression and apoptosis inhibition. 

## 4. Materials and Methods

### 4.1. Cell Lines and Culture

Chinese Hamster Ovary (CHO) cell line (CHO-K1) was purchased from ATCC (Manassas, VA, USA). CHO-K6 cells (stably overexpressing human HER2) [36], CHO-K13 (stably expressing human HER2) [36], and CHO-HER3 (stably expressing human HER2) [46] were obtained as gifts from Drs. Hitt and Buchholz labs (University of Alberta, Edmonton, AB, Canada). CHO-EGFR (stably expressing human EGFR) was previously generated [71]. The cells were cultured in Dulbecco’s modified Eagle’s medium (DMEM) containing 10% fetal bovine serum (FBS) and maintained at 37 °C in a 5% CO_2_ atmosphere. Transgenic selection was maintained by adding of G418 (200 µg/mL) for CHO-K6, CHO-K13 and CHO-EGFR, and hygromycin (200 µg/mL) for CHO-HER3 to the culture medium. The cells were starved overnight at DMEM containing 1% FBS before the treatments. 

### 4.2. Chemicals and Antibodies

HER2 kinase inhibitor CP-724714 was purchased from Selleckchem (Houston, TX, USA). Pertuzumab (Perjeta^®^) and trastuzumab (Hercepton^®^) were purchased from Hoffmann-La Roche (Basel, Switzerland). Mouse monoclonal anti-human HER2 (9G6) (sc-08), anti-human HER2 (A-2) (sc-393712), anti-human EGFR (A-10) (sc-373746), anti-human HER3 (RTJ.2) (sc-415), and rabbit polyclonal anti-human pY1248 HER2 (sc-12352-R) antibodies were purchased from Santa Cruz Biotechnology Inc. (Dallas, TX, USA). Rabbit polyclonal anti-human pY1005, pY1112, pY1127, pY1139, and pY1196 HER2 were purchased from FroggaBio (Toronto, ON, Canada) and mouse monoclonal anti-human pY1221/1222 HER2 (6B12) (2243) were from Cell Signaling Technology (Danvers, MA, USA). Anti-rabbit and anti-mouse RDye^®^ 800CW and RDye^®^ 650 secondary antibodies were purchased from LI-COR biotechnology Inc. (Lincoln, NE, USA). Isotype control human IgG and all other chemicals were purchased from Sigma-Aldrich (St. Louis, MO, USA).

### 4.3. Western Blotting

Total protein was extracted as described previously [72]. Protein samples were prepared by boiling in half volume of 4X laemmli buffer for 5 min. Twenty micrograms of total protein was run in 8% polyacrylamide gel by vertical electrophoresis at 100 V electric potential for 100 min, and then transferred on nitrocellulose membrane at 15 V electric potential for 90 min using a semi-dry protein transfer system (Bio-Rad Laboratories, Berkeley, CA, USA). The membranes were blocked by incubation in Odyssey^®^ Blocking Buffer (TBS) (LI-COR biotechnology Inc., Lincoln, NE, USA) for 60 min and then were incubated overnight in 0.2 µg/mL primary antibody solution. After washing with Tris-buffered saline (TBS) containing 0.05% tween-20, the membranes were incubated in 25 ng/mL RDye^®^ primary antibody solution for 60 min and then after washing were visualized by using Odyssey^®^ CLx imaging system (LI-COR biotechnology Inc., Lincoln, NE, USA). Query protein bands intensity were quantified and normalized to intensity of relevant loading control protein bands. 

### 4.4. Immunofluorescence Assay

The indirect double-immunofluorescence staining was done as described previously [73]. A number of 10^5^ cells were seeded on 15 mm round cover glass in 24 well-plates and were cultured in standard culture condition for 48 h and then were starved overnight in 1% FBS culture medium. After treatment, the coverslips were washed with ice-cold PBS and the cells were fixed by incubation in −20 °C methanol for 5 min. Then, the coverslips were washed with TBS and were blocked in 1% bovine serum albumin (BSA) solution in TBS for 60 min. After blocking, the coverslips (except those treated with isotype human IgG, pertuzumab and trastuzumab) were incubated in 2 µg/mL of primary antibody for 60 min. The coverslips were washed and then were incubated in 1 µg/mL FITC-conjugated (for anti-HER2 or anti-EGFR or anti-HER3 antibodies) and/or 1 µg/mL rhodamine-conjugated (for pertuzumab) secondary antibodies solutions for 60 min in dark. Afterwards, the coverslips were washed with TBS and then were incubated in 1 µg/mL DAPI solution for 5 min. The coverslips were mounted on microscope slides and were observed under a fluorescence microscope. 

### 4.5. Receptor Dimerization Assay

HER2 receptor dimerization assay was done by using cross-linking reagent as described previously [74]. The cells were cultured in standard culture condition for 48 h and then were starved overnight in 1% FBS culture medium. After treatment, the cells were collected and suspended in 0.5 mL of 1 mM bissulfosuccinimidyl suberate (BS^3^) solution in PBS and were incubated on ice for 60 min for cross-linking reaction. To terminate the reaction 5 µL of 10 mM Tris solution (pH 7.5; approximate final concentration of 0.1 mM) was added and the mixture was incubated on ice for 15 min. After centrifugation at 200× *g* for 3 min, the cross-linking solution was removed, and the cells were lysed by adding 1% NP-40 solution and incubating on ice for 60 min. The lysate was run on 5% polyacrylamide gel and HER2 monomer and homodimer were analyzed by western blotting. 

### 4.6. cDNA Microarray Assay

The cells were plated in 60 mm dishes and starved in RPMA-1640 culture medium containing 1% FBS. After 24 h, the old medium was discarded, and fresh medium supplemented with 10% FBS and containing 10 µg/mL of trastuzumab or pertuzumab or their combination added to the cells and let to culture for 24 h. After the treatment period, the total RNA was isolated using TRIzol reagent (Invitrogen, Waltham, MA, USA) according to standard protocol. Samples for microarray hybridization were prepared according to the Affymetrix Manual Target Preparation for GeneChip^®^ Whole Transcript (WT) Expression Arrays (Affymetrix Inc., Santa Clara, CA, USA). Amount 100 ng of total RNA were used to make double-stranded cDNA following cRNA synthesis. After purification, 15 µg cRNA was subjected to reverse transcription into sense-strand (ss) cDNA when unnatural dUTP residues were incorporated. ss cDNA were purified and 5.5 µg of each ss cDNA was fragmented using uracil DNA glycosylase (UDG) and apurinic/apyrimidinic endonuclease 1 (APE 1) at the unnatural dUTP which breaks the DNA strand. Fragmented ss cDNA were terminal labeled with biotin and 3 µg from each sample was hybridized to Affymetrix GeneChip^®^ CHO Gene 2.0 ST array (format 100) for 16 h at 45 °C with rotation at 60 rpm in an Affymetrix GeneChip^®^ Hybridization Oven 645. After hybridization, the arrays were washed and stained in an Affymetrix GeneChip^®^ Fluidics Station FS450. The fluorescent signals were measured with an Affymetrix GeneChip^®^ Scanner 3000 7G. Row data was analyzed by Affymetrix Transcriptome Analysis Console (TAC) 3.0 software (Affymetrix Inc., Santa Clara, CA, USA) using GeneChip^®^ CHO Gene 2.1 ST Array annotation library (Gene Expression Omnibus (GEO) platform number GPL24076). Normalized microarray cDNA expression values are available from GEO database by accession number GSE110189.

### 4.7. MTT Proliferation Assay

A number of 10^4^ cells were seeded as per 96-well plates and cultured in 100 µL DMEM medium containing 10% FBS. After 24 h culture, medium was replaced with fresh DMEM containing 1% FBS and were left to starve overnight. The starvation medium then replaced with medium supplemented with 10% FBS and containing treatment agent, and the cells were incubated for 72 h. At the end of the treatment time, 10 μL of 12 mM MTT solution (Sigma Aldrich, St. Louis, MO, USA) was added to the wells and then the plates were incubated at 37 °C for 4 h until the blue formazan crystals form. Afterwards, the solution was removed and replaced with 50 μL dimethyl sulfoxide (DMSO). The plates then were incubated at 37 °C for 10 min until the crystals dissolve and blue color develops. The color intensity of was measured at 540 nm wave-length using a microplate reader. The absorbance values were normalized to those of blank wells.

### 4.8. Statistical Analysis

Blot band intensity was qualified by ImageJ software [75]. Hierarchical heatmaps were created by Heatmapper [76]. Data were statistically analyzed by one-way analysis of variance (ANOVA) using Prism V.6 software (GraphPad Software, La Jolla, CA, USA). Data was presented as mean and standard deviation. *p* < 0.05 was considered as statistically significant.

## 5. Conclusions

In conclusion, we show here that pertuzumab does not inhibit HER2 homodimerization and phosphorylation. Instead, pertuzumab induces phosphorylation of HER2 at Y1127, Y1139, and Y1196 phospho-sites independent of HER2 homodimerization. Moreover, pertuzumab did not block HER2 homodimer-induced cell proliferation. These data suggest that the clinical effects of pertuzumab may mostly through the inhibition of HER2 heterodimers, rather than HER2 homodimers. Our data suggest that pertuzumab may suppress HER2 function activated through non-canonical pathway(s) rather than its canonical downstream pathways (PI3K/AKT and MAPK). One of the candidate oncogenic mechanism of HER2 independently of PI3K/AKT and MAPK is proteolytic cleavage of HER2 and production of p95HER2 fragments which is reported correlated with more aggressiveness and poor prognosis of breast cancer. Indeed, we observed the lower bands of HER2 in our Western blots (Figure 1A), which may represent the cleavage of HER2. p95HER2 fragments are C-terminal HER2 proteins with approximately 95 KDa in molecular weight are able to translocate to the nucleus and act as transcription co-factor [77]. Investigating the function of p95HER2 and the role of pertuzumab and trastuzumab in HER2 cleavage and production of p95HER2 is required to better understand biology of HER2-positive breast cancer and the exact mechanism of action of the anti-HER2 monoclonal antibodies. We also introduce CHO-K6 cell line as a suitable in vitro model for studying function of HER2 independently of HER family receptor canonical downstream pathways PI3K/AKT and MAPK.
